# Dental Law in Missouri

**Published:** 1897-07

**Authors:** 


### Dental Law in Missouri.

A letter from a correspondent in St. Louis recently, remarks: "Our State has just succeeded in passing a law creating a Board of Dental Examiners. The Board was appointed last week. Hereafter, we hope that Missouri will not be made a dumping ground for quacks and incompetency as it has been before." It is a matter of gratification that the profession in Missouri has finally succeeded in obtaining a dental law, and it is to be hoped the law will be fully executed, and that the public and profession in Missouri will share the benefits of such a law to the full.

